# Design, Synthesis, and Pharmacological Evaluation of Haloperidol Derivatives as Novel Potent Calcium Channel Blockers with Vasodilator Activity

**DOI:** 10.1371/journal.pone.0027673

**Published:** 2011-11-16

**Authors:** Yicun Chen, Jinhong Zheng, Fuchun Zheng, Jinzhi Wang, Yanmei Zhang, Fenfei Gao, Zhanqin Huang, Ganggang Shi

**Affiliations:** 1 Department of Pharmacology, Shantou University Medical College, Shantou, Guangdong, China; 2 Department of Chemistry, Shantou University Medical College, Shantou, Guangdong, China; 3 Department of Pharmacy, First Affiliated Hospital, Shantou University Medical College, Shantou, Guangdong, China; 4 Department of Cardiovascular Diseases, First Affiliated Hospital, Shantou University Medical College, Shantou, Guangdong, China; Virginia Commonwealth University, United States of America

## Abstract

Several haloperidol derivatives with a piperidine scaffold that was decorated at the nitrogen atom with different alkyl, benzyl, or substituted benzyl moieties were synthesized at our laboratory to establish a library of compounds with vasodilator activity. Compounds were screened for vasodilatory activity on isolated thoracic aorta rings from rats, and their quantitative structure–activity relationships (QSAR) were examined. Based on the result of QSAR, *N*-4-tert-butyl benzyl haloperidol chloride (**16c**) was synthesized and showed the most potent vasodilatory activity of all designed compounds. **16c** dose-dependently inhibited the contraction caused by the influx of extracellular Ca^2+^ in isolated thoracic aorta rings from rats. It concentration-dependently attenuated the calcium channel current and extracellular Ca^2+^ influx, without affecting the intracellular Ca^2+^ mobilization, in vascular smooth muscle cells from rats. **16c**, possessing the *N*-4-tert-butyl benzyl piperidine structure, as a novel calcium antagonist, may be effective as a calcium channel blocker in cardiovascular disease.

## Introduction

Calcium is essential for life and is the most common signal transduction element in cells [1], [2]. The contraction of all types of muscle primarily depends on increased intracellular Ca^2+^ [3]. Calcium channel blockers constitute an important class of cardiovascular drugs. Members of this class are clinically useful in the treatment of cardiovascular disorders in which calcium plays a regulatory role. However, most cannot be used to resolve clinical problems such as ischemia, reperfusion injury, and vascular structure remodeling [4]. Furthermore, some of them have serious side effects. Therefore, better calcium channel blockers are still needed.

**Figure 1:**
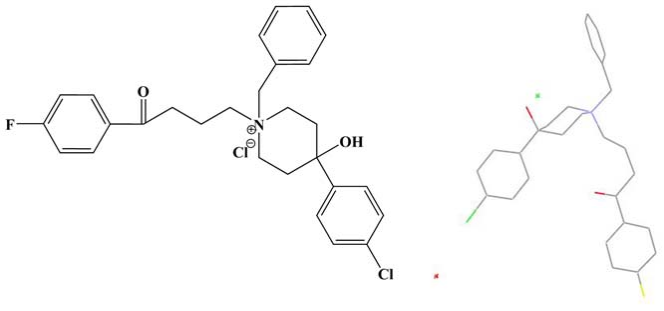
The chemical structure of 4(A), The molecular structure of 4 (B).

Haloperidol is a widely used antipsychotic whose therapeutic properties have been associated with its D_2_ antagonist activity on the basis of the so-called dopamine hypothesis [5]. *N*-Benzyl haloperidol chloride (**4**) (Figure 1A), a derivative of haloperidol, was synthesized at our laboratory and was found to have a vasodilator effect [6]. Our previous study revealed that **4**'s inhibitory effect on the influx of extracellular Ca^2+^ might be related to vasodilatory mechanisms [7]. Furthermore, the *N*-benzyl piperidine structure of **4** is completely different from that of the clinically used calcium channel blockers, which belong to one of the following three chemical classes: the dihydropyridines, the phenylalkylamines, and the benzothiazepines [8]. Therefore, a novel calcium channel antagonist may be obtained by molecular manipulation of the structure of **4**, which itself is not a sufficiently potent vasodilator.

With the aim of exploring the structure–activity relationship of **4** and obtaining more efficient calcium channel blockers, we had cultured single crystals to determine the structure of **4**. Furthermore we designed the series of compounds shown in Table S1. In addition, we revealed some haloperidol derivatives (compounds **6a–6c**, **9a–9c** in Table S1) had vasodilator activity in previous study [9]. The piperidine scaffold, which we consider a supporting structure for novel calcium channel antagonists, was decorated at the nitrogen atom of piperidine with different substituent groups. We screened these compounds for vasodilatory activity in isolated rat thoracic aorta rings held in a Ca^2+^-containing high-K^+^ solution. A detailed quantitative structure–activity relationship (QSAR) analysis of this series of haloperidol derivatives was subsequently performed to study the physicochemical properties that were responsible for their activity. Base on the result of QSAR, we synthesized *N*-4-tert-butyl benzyl haloperidol chloride (**16c**) which was found to posses a good vasodilative activity in KCl-constricted vessels in line with expectation.

Many studies have demonstrated that vasorelaxation in KCl-constricted vessels can be ascribed directly to the inactivation of voltage-dependent Ca^2+^ channels (VDCCs) [10]. Hence, **16c** was further investigated for its inhibitory action on the contraction caused by an influx of exogenous Ca^2+^ in thoracic aorta rings to prove that its vasodilator activity was related to Ca^2+^. Then, the inhibitory effects of **16c** on the activity of VDCCs, the extracellular Ca^2+^ influx, and the intracellular Ca^2+^ release in vascular smooth muscle cells (VSMCs) were explored in detail to reveal the calcium antagonist mechanism.

## Results

### Synthesis

**Figure 2:**
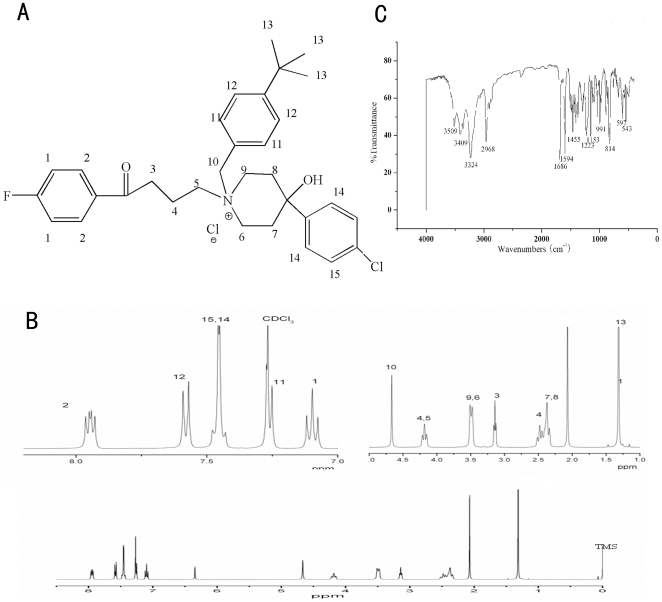
The chemical structure of 16c (A); 400 MHz ^1^H NMR spectrum of 16c in CDCl_3_ (B); Infrared Spectroscopy of 16c (C).

The reaction pathways used to synthesize the designed compounds (compounds **1–16**) were described in Schemes S1, S2, and the chemical and physical characteristics of the compounds were reported in Table S1. The key intermediate haloperidol (4-[4-(4-chlorophenyl)-4-hydroxy-1- piperidyl]-1-(4-fluorophenyl)-butan-1-one) could be obtained via piperidyl alkylation reaction according to the literature [11]. Commercially available alkyl and benzyl halides were used. Some of benzyl halides (compounds **I–V**) were synthesized from the corresponding benzaldehyde compounds (Scheme S1). The quaternary ammonium salt derivatives of haloperidol (compounds **1–16**) were synthesized from haloperidol by alkylation or benzylation at the nitrogen atom using a suitable alkyl or benzyl halide (Scheme S2). In order to know about the structure of **4** as the leading compound clearly, we had cultured single crystals and determined the crystal structure by X-ray single crystal diffraction (Figure 1B, Table S2). The chemical structures of these compounds were characterized by ^1^H NMR spectra (Table S3). **16c** was illustrated as a representation in Figure 2.

### Determination of vasorelaxation

All synthesized compounds caused a concentration-dependent relaxation of aortic rings that had been precontracted to varying extents with a high level of K^+^ (80 mM). The vehicle (0.1% DMSO) used for all compounds had no effect on vascular tone. Concentration–response curves were determined for each compound, and the potency was expressed as the IC_50_ value in the presence and absence of endothelium (Table S4). From Table S4, we can see no significant IC_50_ difference between aortic rings with intact endothelium or denuded endothelium. Concentration-response curves showed no significant shift in the absence of endothelium, than in the presence of endothelium, which suggested no nitric oxide involvement in the vasodilatation (data not shown).

#### QSAR study of some derivatives

A detailed QSAR analysis of a series of haloperidol derivatives (compounds **4–15**, splitting into a testing set of 3 compounds which are randomly selected and training set of 26 compounds) had been performed to determine the physicochemical properties responsible for their activity. The physicochemical parameters used were listed in Table 1, namely the hydrophobic constant π, Hammett σ, molar refractivity (MR), and were compiled from the literature [12]–[14]. The IC_50_ values were transformed to pIC_50_ (negative logarithm of IC_50_). The QSAR results indicated that activity was best modeled by molecular steric parameters represented by MR, hydrophobic parameters represented by π. The best QSAR obtained was:
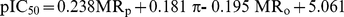



**Table 1:** Physicochemical properties and lgIC**_50_** of the substituent.

Compound	π	σ	MR_o_ #	MR_m_ #	MR_p_ #	lgIC_50_ (M)* Experimental	lgIC_50_ (M)* Predicted
**4**	0	0	0.1	0.1	0.1	−4.9965	−5.06832
**5a**	−0.67	−0.2	0.28	0.1	0.1	−4.8877	−4.91286
**5b**	−0.67	0.12	0.1	0.28	0.1	−4.9597	−4.94857
**5c**	−0.67	−0.37	0.1	0.1	0.28	−4.9974	−4.99122
**6a**	0.71	0.40	0.6	0.1	0.1	−5.07	−5.09602
**6b**	0.71	0.37	0.1	0.6	0.1	−5.2097	−5.19522
**6c**	0.71	0.23	0	0.1	0.6	−5.3001	−5.33352
**7b**	0.64	0.39	0.1	1.25	0.1	−5.0074	−5.18271
**7c**	0.64	0.31	0.1	0.1	1.25	−5.2111	−5.45515
**8a**	0.88	0.97	0.5	0.1	0.1	−5.0301	−5.14624
**8b**	0.88	0.64	0.1	0.5	0.1	−5.3595	−5.22561
**8c**	0.88	0.56	0.1	0.1	0.5	−5.44	−5.32037
**9a**	0.56	0.93	0.56	0.1	0.1	−5.07	−5.07715
**9b** &	0.56	0.8	0.1	0.56	0.1	−5.1999	−5.16814
**9c**	0.56	0.73	0.1	0.1	0.56	−5.27	−5.27739
**10a**	0.14	0.98	0.09	0.1	0.1	−5.02	−5.09533
**10b**	0.14	0.92	0.1	0.09	0.1	−5.0798	−5.09335
**10c**	0.14	0.93	0.1	0.1	0.09	−5.07	−5.09098
**11a**	−0.28	1.03	0.74	0.1	0.1	−4.9698	−4.8913
**11b** &	−0.28	0.86	0.1	0.74	0.1	−5.1402	−5.01778
**11c**	−0.28	0.73	0.1	0.1	0.74	−5.27	−5.1699
**12c**	1.53	−0.15	0.1	0.1	1.50	−5.6458	−5.67345
**13b**	−0.01	0.36	0.1	1.29	0.1	−5.266	−5.06654
**13c**	−0.01	0.45	0.1	0.1	1.29	−5.4485	−5.34845
**14a**	−0.57	1.28	0.63	0.1	0.1	−4.7201	−4.86129
**14b**	−0.57	1.07	0.1	0.63	0.1	−4.9299	−4.96587
**14c** &	−0.57	0.91	0.1	0.1	0.63	−5.0899	−5.08827
**15a**	1.02	−0.17	1.03	0.1	0.1	−5.1598	−5.06611
**15c**	1.02	−0.15	0.1	0.1	1.03	−5.58	−5.47095
**16c**	1.98	−0.02	0.1	0.1	1.96	−6.0222	−5.86636

*, IC_50_: The half maximal inhibitory concentration,

# , MR_o_: molar refractivity in ortho-position, MR_m_: molar refractivity in meta-position, MR_p_: molar refractivity in para-position.

& , compounds as testing set and their predicted IC_50_ were calculated by QSARs model.

**Figure 3:**
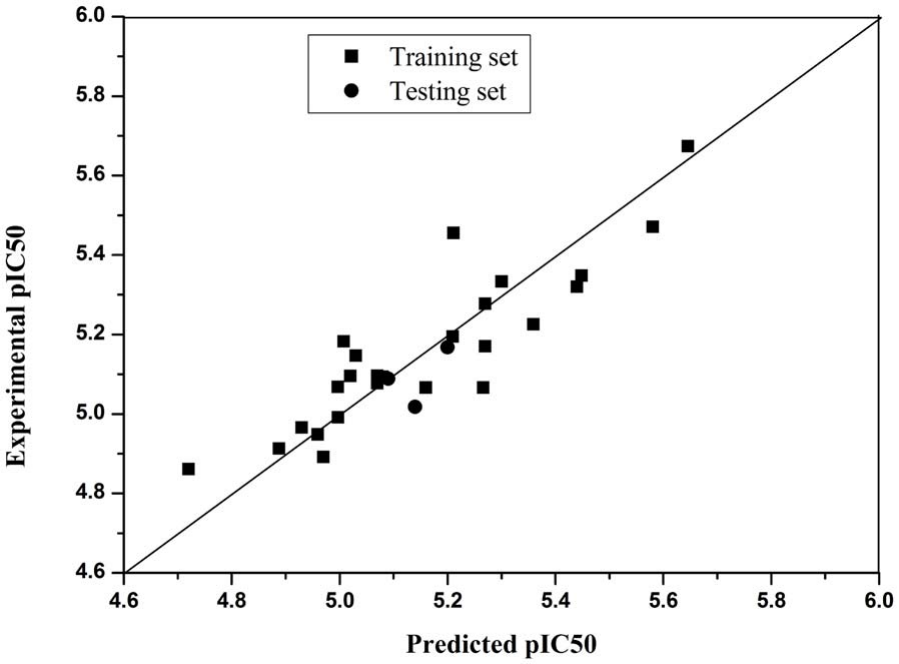
Correlation between predicted activities (IC_50_) by QSARs models and the experimental activities (IC_50_) (for compounds of training set, R^2^ = 0.787; for randomly selected compounds 9b,11b,14c as testing set, Residue are 0.03176; 0.1224, 0.00163. **Correlation is significant at the 0.01 level.).**

The subscripts in the parameter relate to the position of the substituent considered. A determinants of coefficient R^2^ is 0.787 for 26 compounds in training set, and the residuals of compounds 9b,11b,14c as testing set are 0.03176; 0.1224, 0.00163. It confirmed that the equation was of some value in predicting activity (Table 1, Figure 3).

### The inhibitory action of 16c on the contraction caused by KCl and the influx of extracellular Ca^2+^

The results indicated **16c** showed the most significant vasorelaxation effect on KCl-dependent contraction compared with compounds 1–15, and the IC50 value of **16c** was 0.95 µM (Table 1). And 16c was chosen for further research.

**Figure 4:**
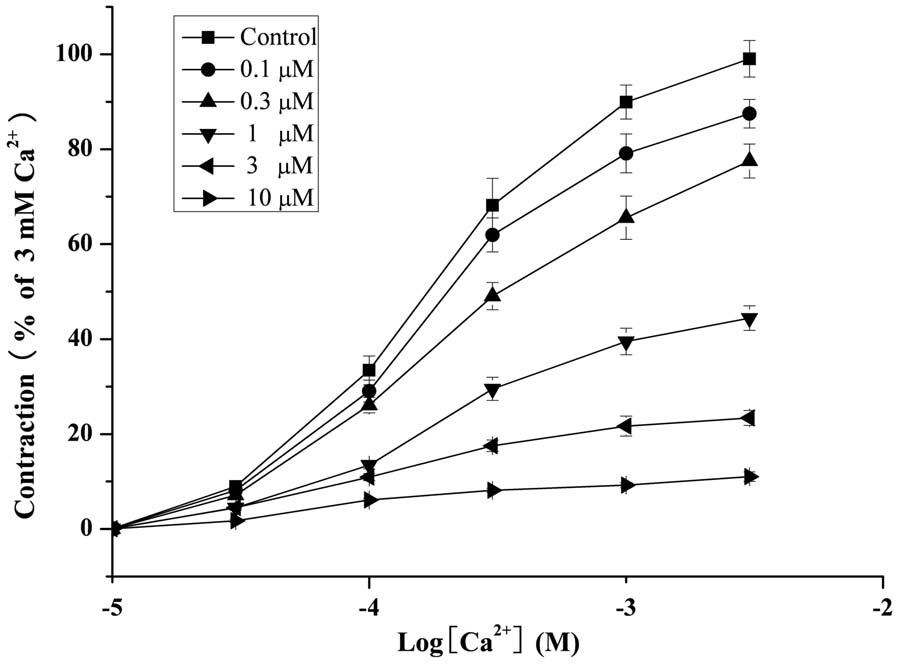
Cumulative concentration-response curves for Ca^2**+**^ in the presence of the vehicle and increasing concentrations of 16c (0.1–10 **µ**M) in isolated endothelium-intact aortic rings (n = 5). Results are the mean ±S.E. from 5 independent experiments.

KCl-dependent contraction is due to the influx of extracellular Ca^2+^ through VDCCs [10]. We first investigated the ability of **16c** to inhibit the contraction induced by the exogenous application of Ca^2+^ in a Ca^2+^-free high-K^+^ solution. As Figure 4 showed, **16c** at different concentrations (0.1—10 µM) significantly impaired the response to Ca^2+^ (3 mM) to 87.49%—11.02% of the initial response. The IC_50_ value was 0.76 µM, similar to the result obtained from the determination of vasorelaxation (Table S1, Table 1). The results indicated that **16c** inhibited the responses to Ca^2+^ in a concentration-dependent manner. So we deduced that **16c** significantly inhibited the response to Ca^2+^ influx from the extracellular space.

#### Effects of 16c on [Ca^2+^]_i_ in arterial VSMCs

**Figure 5:**
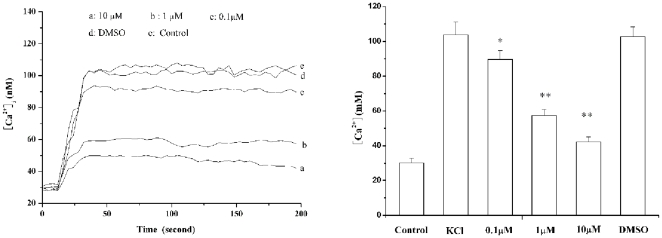
Effect of 16c (0.1∼10 **µ**M) and the vehicle (0.1% DMSO) on KCl (80 mM)-induced changes of Ca^2+^ concentration in vascular smooth muscle cells. A, representative Ca^2+^ concentration response to KCl ; B, dose-dependent inhibitory effect of **16c** on the Ca^2+^ concentration response to KCl. Results are the means ± S.E. (n = 24). *, *p*<0.01 compared with normal. **, *p*<0.01 compared with model (KCl).

The Ca^2+^ responses of arterial VSMCs to KCl (80 mM) were studied after incubation with **16c**. **16c** did not affect basal [Ca^2+^]_i_ (29.8±1.2 *vs* 30.7±1.4 nM for **16c** and control, respectively, *n* = 25, *p*>0.05). The response of the control cells to KCl was characterized by a sharp increase in [Ca^2+^]_i_ (from 30.18±2.82 to 103.34±7.26 nM, mean ±S.E. *n* = 24) followed by a sustained plateau (106.64±6.24 nM at 200 s). This response was totally inhibited by **16c** at 0.1, 1, 10 µM (both KCl ^peak^ and KCl ^200 s^ = 89.76±4.96, 57.24±3.64, 42.32±2.65 nM, respectively *n* = 24, *p*<0.01 *vs* control values; Figure 5).

#### Effect of 16c on L-type Ca^2+^ channel activity

**Figure 6:**
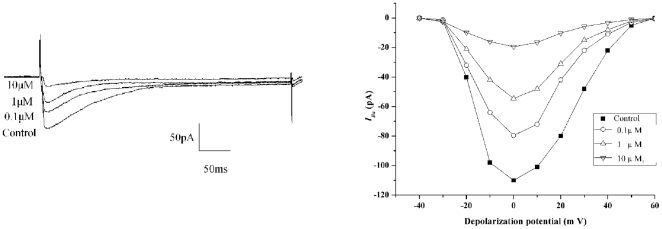
Effect of 16c on L-type Ca^2+^ channel currents in vascular smooth muscle cells (A) and on *I–V* relation of *Ba^2+^* currents in vascular smooth muscle cells (B). The step protocol of recording was described in methods section.

We then applied the patch-clamp technique to measure the effect of **16c** on the activity of VDCCs on the primary cells. In our study, *I*_Ba_ was elicited by depolarization from the depolarizing potential of −30 mV to +50 mV. The peak *I*_Ba_ was elicited at the potential of 0 mV. Barium currents were reversibly inhibited by 1 µM nimodipine, and augmented by 50 nM Bay K 8644 (data not shown). **16c** (10, 1, 0.1 µM) reduced the peak current to 21.3±5.1% (*p*<0.01, n = 5), 39.4±6.2% (p<0.01, n = 5), 89.8±15.1% (p<0.01, n = 5) of control values, respectively (Figure 6A). The VSMCs showed current/voltage relationships typical of high voltage-activated L-type Ca^2+^ channels, with apparent threshold and reversal potentials of approximately −30 and +50 mV, respectively (Figure 6B). No change in leak current was observed during the application of the compounds, which suggests that at these concentrations, the compounds had no “detergent-like” effect on cell plasma membrane. Half-maximal inhibition of the Ca^2+^ channel current was observed at about 1 µM.

## Discussion

Calcium channel blockers play an important role in cardiovascular diseases, but better drugs are still needed for some clinical problem. In our study, we found some Haloperidol derivatives showed vasorelaxation activity to varying degree. We synthesized a series of haloperidol derivatives and we used the test of vasodilator effect on the rat isolated thoracic aorta rings with a high level of K^+^ (80 mM) to screen molecules for further studies, and examined the structure-activity relationship of compounds. Studies have demonstrated that the contraction of vascular smooth muscle is initiated by an increased intracellular calcium level [15]–[17], which may be achieved in two ways: extracellular Ca^2+^ influx from VDCCs evoked by depolarization with high potassium concentration, and intracellular Ca^2+^ release from the intracellular stores [18], [19]. From the result, we can deduce these compounds' inhibition on the contraction of the vascular smooth muscle might relate to the extracellular Ca^2+^ influx from VDCCs evoked by depolarization with high K^+^.

Furthermore, most published QSARs show the importance of particular physicochemical parameters in describing activity [20]. We observed a correlation between the pharmacological activities and structures in this study. The best QSAR obtained was: pIC_50_ = 0.238 MR_p_+0.181 π- 0.195 MR_o_+5.061. This result showed that the main factors governing activity were the MR term of the specific position of the substituent which will determine the fit at the receptor site and the hydrophobic parameters which determine the ability of the compounds to transport the cellular membrane and to bind in the hydrophobic space of the proteins in the cellular membrane. In addition, compound 4 displayed higher vasodilation activity than compounds 1–3 (from Table S1). These results also indicated that for this series of derivatives, the MR term of a particular molecule was the significant element of the substituent. The importance of both the substituent steric parameters and their position appears to be in their ability to maintain the molecule in an orientation that is conducive to receptor binding. This was inferred from the fact that MR term, as a steric parameter was consistently expressed in a regression analysis. The equation also highlighted the importance of the substituent for vasodilator activity in the ortho- and para-positions. The MR parameter needs some discussion at this point. MR has been viewed as a measure of bulk and as a “rough and ready” steric parameter. It is used to model the intermolecular effects between a ligand and receptor. From the x-ray structure of 4 (Figure 1B), it is easy to understand that the negative ortho effect may be due to a conformational preference of 4-phenyl piperidine ring for the substituted benzyl group. It is, therefore, most probably due to direct steric hindrance interfering with drug receptor binding [21]. Although a more complex problem involving, interpretation of a positive coefficient with MR revealed that the steric effect at the para-position of the aryl ring was advantageous to the activity. In summary, the result showed activity may be further improved if substituents with greater hydrophobic parameters in the para-position are considered (such as p-C (CH_3_)_3_, p-(CH_2_)_4_N(CH_3_)_3_^+^, p-(CH_2_)_4_C_6_H_5_). The compound with p-(CH_3_)_3_ had a lower molecular weight than the others (such as p-(CH_2_)_4_N(CH_3_)_3_^+^, p-(CH_2_)_4_C_6_H_5_). So this compound (16c) was designed and synthesized, and 16c showed the most potent vasorelaxation effect agreeing with the predicted effect (experimental IC_50_: 0.95 µM, predicted IC_50_: 1.36 µM, Table 1).

As KCl-dependent contraction is due to the influx of extracellular Ca^2+^ through VDCCs [10], it is likely that the haloperidol derivatives, such as 16c, may have effects on the inhibition of the influx extracellular Ca^2+^, and the blockade of calcium channels. The ability of 16c to inhibit the contraction induced by the exogenous application of Ca^2+^ then was confirmed in a Ca^2+^-free high-K^+^ solution. This observation suggests that C_3_ might further inhibit the influx of Ca^2+^ through VDCCs.

An elevated [Ca^2+^]_i_ level is the main trigger for VSMC contraction [22], and we showed that 16c had an inhibitory effect on the contraction induced by exogenous Ca^2+^. To reveal the molecular mechanism by which 16c inhibited vasoconstriction and to obtain more information about the effect of 16c on calcium ions, we studied the effect of 16c on Ca^2+^ signaling in VSMC by LSCM. The increase in [Ca^2+^]_i_ is believed to result from Ca^2+^ influx, corresponding to the opening of the voltage-sensitive plasma membrane Ca^2+^ channels [23]. This response was inhibited by 16c, which is consistent with the results in intact arteries (Figure 4). The results of the effect on KCl-dependent Ca^2+^ signaling indicated that 16c decreased the influx of extracellular Ca^2+^ caused by depolarization in VSMCs.

Based on these findings, we therefore postulate that the compounds targeted the plasma membrane, especially the VDCCs present in VSMCs. The patch-clamp technique was applied to measure the effect of 16c on the activity of VDCCs on the primary cells. 16c obviously suppressed the calcium channel current and the I-V relation of *I_Ba_* indicated that the blocking effect of 16c on calcium channels was voltage-independent. Studies indicate two types (L and T) of calcium channel exist in VSMCs [24]. Under the condition of individual cell depolarization from a holding potential of −40 mV, the L-type calcium channel was activated, and the T-type Ca^2+^ and Na^+^ channels were inactivated. Moreover, TEA, a non-specific K channel blocker, was administered in the extracellular solution. The pipette solution was also filled with 4-AP (a K^+^ channel blocker). So the outward K^+^ currents were completely blocked [25]. In addition, Barium currents were reversibly inhibited by 1 µM nimodipine, and augmented by 50 nM Bay K 8644, an activator of L-type Ca^2+^ channels [26] in our study. Therefore, the inward current we recorded under these conditions was an L-type Ca^2+^ current. Obviously, 16c suppressed the L-type calcium channel current. In addition, we can infer the mechanism of 16c inhibition of L type Ca^2+^ channels, from the structure of 4-phenyl piperidine which exists in the haloperidol derivatives. In our study, the structure-activity relationship analysis indicated the 16c and other haloperidol derivatives, which contain 4-phenyl piperidine ring possess calcium channel blocking (CCB) activity and the conformation of the 4-phenyl piperidine ring correlates with the CCB activity. The piperidine ring is the hydride 1, 4-DHP ring. Studies on the structure of nifedipine demonstrated the 1, 4-dihydropyridine (1, 4-DHP) ring system which was perpendicular to the C-4 substituted-phenyl ring, was the determinant of calcium channel antagonist activity [27]. Therefore we suppose the mechanism of 16c in the block of L-type Ca^2+^ channel is probably similar to the nifedipine. The heterologous expressing such as Xenopus oocytes, will be performed to explore the exact mechanism in our future studies.

Ca^2+^ is considered to be an intracellular signal implicated in both vasoconstriction and proliferation [28]. However, it is well known that some L-type Ca^2+^ channel antagonists(verapamil, diltiazem, and nifedipine)show little inhibitory effect on the proliferation of vascular smooth cells, which is related to the development of vascular diseases [29]. While, it was reported that 16c, a novel channel blocker, had anti-proliferation activity [30]. Ca^2+^, as a second messenger, stimulates nuclear transcription factors Egr-1 expression which involves cell proliferation [30]. We previously showed that Egr-1 overexpression induced cell proliferation by using antisense Egr-1 ODNs, and demonstrated that 16c attenuated the Ang II-induced extracellular Ca^2+^ influx and inhibited Egr-1 overexpression. In this study, we demonstrated further that 16c had vasodilatory activity, although the vasodilator effect of 16c was not more potent than other L-type Ca^2+^ antagonists (nifedipine, 1–10 nM; verapamil, 0.1–1 µM; and diltiazem, 0.5–1 µM; concentrations for 50% inhibition of vascular contractions in rat or rabbit aorta) [31].

However, 16c, as a novel channel blocker, had vasodilatory activity and anti-proliferation activity that provides a basis for further studies of novel calcium channel blockers on intervention of vascular remodeling and atherosclerosis [30].

## Methods

### Chemistry

#### General methods for preparation of the haloperidol derivatives

The preparation of the title compounds was very simple. Haloperidol was dissolved in chloroform and was then reacted with benzyl halide under reflux conditions, or halohydrocarbon (iodomethane, ethyliodide, iodopropane) were used as a solvent and were reacted with haloperidol directly. All melting points were taken on a Büchi apparatus and were uncorrected. IR spectra were recorded with a Nicolet-Impact 410 system as KBr pellets. ^1^H NMR spectra were recorded on a Bruker AM-400 spectrometer (400 MHz). The ^1^H NMR internal standard used was tetramethylsilane (TMS). Chromatographic separations were performed on a silica gel column by gravity chromatography (Kieselgel 40, 0.01–0.04 mm; Merck). Yields are given after purification, unless otherwise stated. Where analyses are indicated by symbols, the analytical results are within ±1% of the theoretical values. All spectral and elemental analysis data (shown in the Supporting Information) were in agreement with the assigned structures. All compounds were estimated to be >95% pure according to analytical reversed-phase high performance liquid chromatography (RP-HPLC) and detection with MS (see Supporting Information for details) for details. No UV-active impurities were observed with analytical RP-HPLC and UV detection at 245 nm. The data for **16c** was displayed as represented in Figure 2.

#### Synthesis of compounds 1–3

Haloperidol (10 mM) was dissolved in the corresponding halohydrocarbon (4 ml). The mixture was heated in an oil bath for 24 h to maintain reflux and was then cooled to room temperature. The filtered solid was recrystallized from an ethanol-water mixed solvent to a crystalline product.

#### Synthesis of some benzyl halide (compounds I–V)

The corresponding benzaldehyde compounds (100 mM) were dissolved in methanol (20 ml)(for I–III) or THF (20 ml) (for IV–V). NaBH_4_ (200 mM) was then added gradually in ice-bath. The mixtures were elevated to room temperature and stirred for 5 min(for I–III) or 1 h (for IV–V). Then water (20 ml) was added to end the reaction and the mixtures were evaporated to dryness. The products were again dissolved in ethylacetate (40 mL), washed with water (20 ml) and ethylacetate extractions were concentrated to dryness. The products of benzyl alcohol derivatives were obtained in this step. These products were then dissolved in THF (20 ml without water, contained DMF 1–2 d), SOCl_2_ (3 ml) was added gradually in ice-bath. After the mixtures were stirred at 0°C for 20 min, reactions were terminated and the pH value of the mixture was adjusted to about 7 with a 1 N NaOH solution. The oil layers were separated from the mixture with a separatory funnel and the products (benzyl halide) were obtained by distillation. The ^1^HNMR data of compounds **I–V** were compared with literature [32], [33], and reported in supporting information (Table S5).

Others benzyl halide were purchased from *Alfa Aesar*, *A Jhnson Matthey Company* and some Chinese Companies. These compounds have been confirmed with literature data in HNMR.

#### Synthesis of compounds 4–16

Haloperidol (10 mM) was dissolved in chloroform (4 mL) and the corresponding benzyl halide (30 mM) was then added. The mixture was heated to maintain reflux for 18–24 h, and was then cooled to room temperature. The white solid was filtered off, and was washed with chloroform. The crude product was recrystallized in an ethanol–water mixed solvent to provide a crystalline product.

### Pharmacology

#### Vasoactivity determination

The vasodilation activity of compounds **1–16** was evaluated in isolated rat thoracic aortic rings according to the methods of Towart and Polster et al [34], [35]. Thoracic aortas were isolated from male Wistar rats weighing 200–250 g. The vessels were cut into ring segments, 2–3 mm wide, and 5 rings were obtained from each aorta. Rings were then mounted in standard 10-mL organ baths filled with Krebs bicarbonate solution ((PSS), containing (in mM): 118.0 NaCl, 4.7 KCl, 1.2 KH_2_PO_4_, 1.2 MgSO_4_.7H_2_O, 5.0 glucose, 25.0 NaHCO_3_, and 2.5 CaCl_2_.7H_2_O) at 37°C and were gassed with 95% O_2_–5% CO_2_. (pH = 7.4). The aortic rings were allowed to equilibrate for 90 min at a testing tension of 1 g, and were then depolarized by the addition of a solution of KCl to a final concentration of 80 mM. The preparations were then extensively washed with PSS, and a second contraction was evoked by K^+^ depolarization (80 mM). When the amplitude of the contraction reached a plateau, cumulative concentrations of the tested compounds in a vehicle of water and DMSO (1000∶1) were added every 45 min. Development of antagonism occurred so slowly that doses had to be increased at established times, without waiting for complete equilibrium to be reached. Cumulative concentration-response cures to all compounds were determined in the absence and presence of endothelium. According to experimental protocol, when required, the endothelium was mechanically removed from some rings by gently rubbing the lumen with the tips of a forceps. Acetylcholine was used to confirm that the endothelium had effectively been removed from the aortic rings [36].

#### Inhibitory action of active compound (16c) on the contraction caused by influx of extracellular Ca^2+^

To determine the effect of **16c** on the response to the influx of extracellular Ca^2+^, the contractile response to the exogenous application of Ca^2+^ was examined in the presence of **16c** in Ca^2+^-free high-K^+^ solution (80 mM, K^+^-HBSS) as previously described [37]. Aortic rings were initially equilibrated at a resting tension of 1 g in normal Ca^2+^-free HBSS for 60 min. The bath medium was then replaced with Ca^2+^-free high-K^+^ HBSS for 30 min to determine a control response to Ca^2+^ (3 mM) and this was considered to be 100% contraction. After a 30 min equilibration with Ca^2+^-free HBSS, the cumulative contractile response to Ca^2+^ (0.01–3 mM) was determined in K^+^-HBSS in the presence of increasing concentrations (0.1–10 µM) of **16c**. The aortic rings were incubated with **16c** for 10 min before examining the responses to Ca^2+^.

#### Electrophysiology

Inward barium (***I***_Ba_) currents were studied in single cells by the patch-clamp technique in the perforated-attached configuration [38]. The recordings were made using a Biologic RK-300 amplifier, filtered (23 dB, 5-pole Tchebicheff filter) at 1 kHz and sampled at 5 kHz. Currents were recorded from holding potential −40 mV during linear voltage ramps from −40 to +60 mV, with 10 mV increments. The bath solution contained (in mM) NaCl 125, BaCl_2_ 10.8, MgCl_2_ 1, CsCl 5.4, glucose 10, and Hepes 10 (pH = 7.4 at 24°C). Patch pipettes were pulled from glass capillaries (Warner Instruments) and were coated with wax to minimize capacitative transients and noise; the electrode resistance ranged from 2–5 MΩwhen filled with the pipette solution. The patch pipette was filled with a solution containing (in mM) CsOH 75, CsCl 55, MgCl_2_ 5, aspartic acid 75, Hepes 10 (pH = 7.4 at 22°C). Nystatin (Biochrom KG, Berlin, Germany) was dissolved in Me_2_SO and diluted into the pipette solution to give a final concentration ranging from 50 to 100 mg/mL. Drugs were applied by changing the bath solution. The final concentration of ethanol, the diluent, was 0.1% (v/v). The sampling and data analysis involved the use of pClamp 8.1 software (Axon Instruments). Experiments were carried out at room temperature (25°C).

#### Isolation of rat primary VSMCs

Primary cell cultures of VSMCs were established with cells removed from abdominal aortas excised from 4-week-old Wistar rats. Isolation of aortic VSMCs followed the method of Campbell et al [39]. The cells were suspended in a growth medium (DMEM supplemented with 10% (v/v) fetal bovine serum [FBS] (JRH Biosciences, Lenexa, KS, USA), 2.2 g/L NaHCO_3_, 100 U/mL penicillin G, and 100 µg/mL streptomycin) and seeded into 24-mm glass coverslips in 6-well plates (for LSCM) or a 0.5-mL chamber (for patch-clamp). Cells were cultured at 37°C in a humidified atmosphere of 5% CO_2_ in air. The experimental protocol was approved by the University Research Committee, and the animal protocol was in full compliance with the *Guidelines for Animal Experimentation* developed by the university.

#### Determination of intracellular free [Ca^2+^]_i_

[Ca^2+^]_i_ was determined in adherent cells after 60 min of loading with 20 µM Fluo3-AM in HBSS, as described previously [40]. Fluorometric data were obtained by means of LSCM (Zeiss LSM 510 META). The Fluo-3 dye was excited with a 488-nm wavelength argon laser beam and the emission fluorescence was monitored at 525 nm. [Ca^2+^]_i_ was calculated according to the method of Grynkiewicz, et al [41]. At the end of the experiment, Triton X-100 and, subsequently, EGTA were added at final concentrations of 0.1% and 1 mM, respectively, to obtain the fluorescence maximum (F_max_) and the fluorescence minimum (F_min_). [Ca^2+^]_i_ was determined from the equilibrium equation, [Ca^2+^]_i_ = K_d_ (F−F_min_)/(F_max_−F), where F is the experimental value of fluorescence and K was defined as 0.4 µM. [Ca^2+^]_i_ was measured at base line first. After the cells were treated with **16c** for 10 min, the agonists, KCl and TG, were added and [Ca^2+^]_i_ was determined.

#### Analysis of statistics

Data were presented as mean ± S.E. Group differences were analyzed by Student's *t* test. The effect of **16c** on currents was evaluated by Student's paired *t-test*. *P*<0.05 was considered statistically significant.

### Associated content

#### Ethics Statement

All animal work was performed according to the international animal welfare guidelines, and protocols were approved by Shantou University Medical College Institutional Animal Care and Use Committee (permit numbers: SUMC2009-010).

## Supporting Information

Table S1 **Designed compounds 1–16 and their sensitivity (IC_50_) in endothelium-intact thoracic aorta rings from rats.** IC_50_: The half maximal inhibitory concentration.
(DOC)

Table S2 **Crystal data and structure refinement for compound 4.**
(DOC)

Table S3 **^1^HNMR data, EI-MS data of Compounds 1–16.**
(DOC)

Table S4 **A comparison of the sensitivity (IC_50_) to different compounds in endothelium-intact and -denuded thoracic aorta rings from rats.**
(DOC)

Table S5 **^1^HNMR data of Compounds I–V.**
(DOC)

Scheme S1 **Reagents and conditions.** (a) NaBH_4_, MeOH, room temperature 5 min, or NaBH_4_, THF, room temperature 1 hour. (b) SOCl_2,_ THF/DMF, ice-bath 20 min.
(TIF)

Scheme S2 **Reagents and conditions.** (a) CH_3_I or CH_3_CH_2_I or CH_3_ (CH_2_)_2_I, reflux, 24 h. (b) The benzyl halidel, CHCl_3_, reflux, 18–24 h.
(TIF)
