# Supplementary material for: Design, Synthesis, and Pharmacological Evaluation of Haloperidol Derivatives as Novel Potent Calcium Channel Blockers with Vasodilator Activity
Source: PLoS One. 2011 Nov 16;6(11):e27673. doi: 10.1371/journal.pone.0027673 (PMC3218019; doi:10.1371/journal.pone.0027673)
Supplement: Table S1 — Designed compounds 1–16 and their sensitivity (IC50) in endothelium-intact thoracic aorta rings from rats. IC50: The half maximal inhibitory concentration. (DOC) [file pone.0027673.s001.doc]

Table S1. Designed compounds 1-16 and their sensitivity (IC_50_) in endothelium-intact thoracic aorta rings from rats.


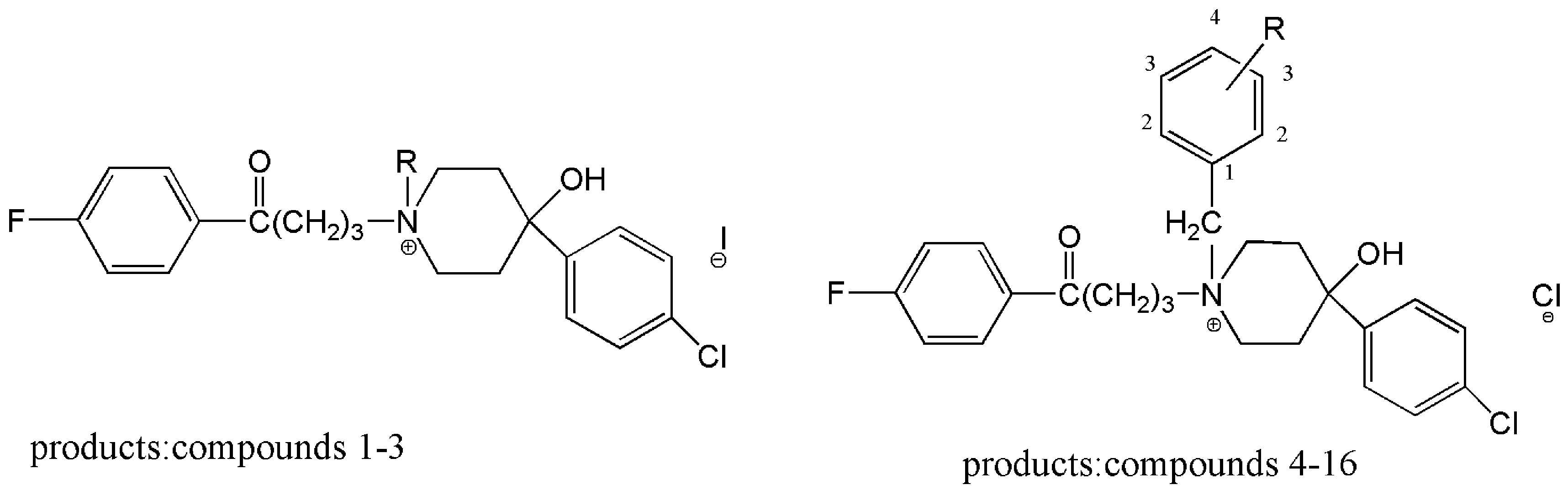


| **Compound** | **R** | **Molecular formula** | **IC_50_** **(μM)** |
| --- | --- | --- | --- |
| **1** | CH_3_ | C_22_H_26_ClIFNO_2_ | 105.02±4.8 |
| **2** | CH_2_CH_3_ | C_23_H_28_ClIFNO_2_ | 32.25±1.9 |
| **3** | (CH_2_)_2_CH_3_ | C_24_H_30_ClIFNO_2_ | 20.2±1.2 |
| **4** | H | C_28_H_30_Cl_2_FNO_2_ | 10.08±0.96 |
| **5a** | 2-OH | C_28_H_30_Cl_2_FNO_3_ | 12.95±1.21 |
| **5b** | 3-OH | C_28_H_30_Cl_2_FNO_3_ | 10.97±0.99 |
| **5c** | 4-OH | C_28_H_30_Cl_2_FNO_3_ | 10.06±1.03 |
| **6a** | 2-Cl | C_28_H_29_Cl_3_FNO_2_ | 8.51±0.82 |
| **6b** | 3-Cl | C_28_H_29_Cl_3_FNO_2_ | 6.17±0.47 |
| **6c** | 4-Cl | C_28_H_29_Cl_3_FNO_2_ | 5.01±0.41 |
| **7b** | 3-OC=OCH_3_ | C_30_H_32_Cl_2_FNO_4_ | 9.83±0.85 |
| **7c** | 4-OC=OCH_3_ | C_30_H_32_Cl_2_FNO_4_ | 6.15±0.52 |
| **8a** | 2-C(F)_3_ | C_29_H_29_Cl_2_F_4_NO_2_ | 9.33±0.74 |
| **8b** | 3-C(F)_3_ | C_29_H_29_Cl_2_F_4_NO_2_ | 4.37±0.32 |
| **8c** | 4-C(F)_3_ | C_29_H_29_Cl_2_F_4_NO_2_ | 3.63±0.22 |
| **9a** | 2-CH_3_ | C_29_H_32_Cl_2_FNO_2_ | 8.51±0.83 |
| **9b** | 3-CH_3_ | C_29_H_32_Cl_2_FNO_2_ | 6.31±0.42 |
| **9c** | 4-CH_3_ | C_29_H_32_Cl_2_FNO_2_ | 5.37±0.61 |
| **10a** | 2-F | C_28_H_29_Cl_2_F_2_NO_2_ | 9.55±0.78 |
| **10b** | 3-F | C_28_H_29_Cl_2_F_2_NO_2_ | 8.32±0.68 |
| **10c** | 4-F | C_28_H_29_Cl_2_F_2_NO_2_ | 8.51±0.72 |
| **11a** | 2-NO_2_ | C_28_H_29_Cl_2_FN_2_O_4_ | 10.72±0.88 |
| **11b** | 3-NO_2_ | C_28_H_29_Cl_2_FN_2_O_4_ | 7.24±0.63 |
| **11c** | 4-NO_2_ | C_28_H_29_Cl_2_FN_2_O_4_ | 5.37±0.42 |
| **12c** | 4-CH(CH_3_)_2_ | C_31_H_36_Cl_2_FNO_2_ | 2.26±0.53 |
| **13b** | 3-C=O(OCH_3_) | C_30_H_32_Cl_2_FNO_4_ | 5.42±0.62 |
| **13c** | 4-C=O(OCH_3_) | C_30_H_32_Cl_2_FNO_4_ | 3.56±0.41 |
| **14a** | 2-CN | C_29_H_29_Cl_2_FN_2_O_2_ | 19.05±1.36 |
| **14b** | 3-CN | C_29_H_29_Cl_2_FN_2_O_2_ | 11.75±1.02 |
| **14c** | 4-CN | C_29_H_29_Cl_2_FN_2_O_2_ | 8.13±0.68 |
| **15a** | 2-CH_3_CH_2_ | C_30_H_34_Cl_2_FNO_2_ | 6.92±0.46 |
| **15c** | 4-CH_3_CH_2_ | C_30_H_34_Cl_2_FNO_2_ | 2.63±0.28 |
| **16c** | 4-C(CH_3_)_3_ | C_32_H_38_Cl_2_FNO_2_ | 0.95±0.072 |

*, IC_50_: The half maximal inhibitory concentration
