# Supplementary material for: Design, Synthesis, and Pharmacological Evaluation of Haloperidol Derivatives as Novel Potent Calcium Channel Blockers with Vasodilator Activity
Source: PLoS One. 2011 Nov 16;6(11):e27673. doi: 10.1371/journal.pone.0027673 (PMC3218019; doi:10.1371/journal.pone.0027673)
Supplement: Table S2 — Crystal data and structure refinement for compound 4. (DOC) [file pone.0027673.s002.doc]

Table S2. Crystal data and structure refinement for compound 4.

| Compound reference | compound 4 |
| --- | --- |
| Chemical formula | C_28_H_30_ClFNO_2_•H_2_O•Cl |
| Formula Mass | 520.45 |
| Crystal system | Triclinic |
| *a*/Å | 9.7107(12) |
| *b*/Å | 10.9817(14) |
| *c*/Å | 13.3548(17) |
| *α*/° | 106.835(2) |
| *β*/° | 100.504(2) |
| *γ*/° | 98.672(2) |
| Unit cell volume/Å^3^ | 1308.5(3) |
| Temperature/K | 293(2) |
| Space group | *P*1 |
| No. of formula units per unit cell, *Z* | 2 |
| No. of reflections measured | 9454 |
| No. of independent reflections | 4572 |
| *R_int_* | 0.0154 |
| Final *R_1_* values (*I* > 2*σ*(*I*)) | 0.0500 |
| Final *wR*(*F*^2^) values (*I* > 2*σ*(*I*)) | 0.1285 |
| Final *R_1_* values (all data) | 0.0604 |
| Final *wR*(*F*^2^) values (all data) | 0.1415 |
