# Supplementary material for: Design, Synthesis, and Pharmacological Evaluation of Haloperidol Derivatives as Novel Potent Calcium Channel Blockers with Vasodilator Activity
Source: PLoS One. 2011 Nov 16;6(11):e27673. doi: 10.1371/journal.pone.0027673 (PMC3218019; doi:10.1371/journal.pone.0027673)
Supplement: Table S3 — 1HNMR data, EI-MS data of Compounds 1–16. (DOC) [file pone.0027673.s003.doc]

Table S3. ^1^HNMR data、EI-MS data of Compounds 1-16

| Compounds | ^1^HNMR/300 MHz, CDCl_3_-d_1_/DMSO-d_6_ | EI-MS (m/z) |
| --- | --- | --- |
| **1** | 1.76-1.79(m,2H,CCH_2_C);2.08-2.15(m,2H,NCCH_2_CCH_2_  C);2.31-2.39(m,2H,COCH_2_); 3.15-3.21 (m,3H,NCH_3_; m, 2H,COCH_2_);3.41-3.58((m,4H,N(CH_2_C)2; m,2H,COCCCH_2_);5.63(s,1H,OH);7.36-7.44(m,4H,Ar-H);7.62-7.64(m,2H,Ar-H);8.05-8.09(m,2H,Ar-H) | 392(M+2),  390(M+) |
| **2** | 1.25-1.36(t,3H,CH_3_);1.73-1.79(m,2H,CCH_2_C);1.96-2.08  (m,2H,NCCH_2_);2.29-2.38(m,2H,NCCH_2_);3.18-3.21(m,2H,COCH_2_);3.38-3.53(m,8H,N(CH_2_)_4_;5.64(S,1H,OH);7.37-7.46(m,2H,Ar-H);7.61-7.66(m,2H,Ar-H),8.058-8.093(m,2H,Ar-H) | 406(M+2),  404(M+) |
| **3** | 0.95-1.02(m,3H,CCH_3_);1.70-1.84(m,2H,CCH_2_CN;m,2H,CCCH2C);1.96-2.07(m, 2H, NCCH_2_C); 2.33-2.35(m, 2H, NCCH_2_C);3.17-3.29(m,2H,COCH_2_);3.35-3.55(m,8H,N(CH_2_)4);5.63(S,1H,OH);7.37-7.46(m,4H,Ar-H);7.60-7.66(m,2H,Ar-H);8.05-8.09(m,2H,Ar-H) | 420(M+2),  418(M+) |
| **4** | 1.77-1.86(m,2H,CCH_2_C);2.30-2·41(m,4H,NCCH_2_CCH_2_  C); 2.51 (t, 2H, COCH_2_); 3.30-3.68 (m, 8H, (CH_2_)_2_N  (CH_2_)_2_);4.75(s,2H,CH_2_Ph);5.68(S,1H,OH);7.36-7.48  (m,5H,Ar-H);7.54-7.58(m,4H,Ar-H);7.86-7.90(m,4H,  Ar-H) | 468(M+2),  466(M+) |
| **5a** | 1.32(m,2H,COCCHC;);2.19-2.24(m,2H,N(CCH_2_)C);2.58-2.62(m,2H,,NCCH_2_C);2.61-2.667(m,2H,COCH_2_);3.12-3.21(m,4H,N(CH_2_C)2);3.61-3.81(m,2H,COCCCH_2_);4.72(s,2H,N-CH_2_-Ar);5.76(s,H,OH);7.38-7.44(m,4H,Ar-H);758-7.64 (m,4H,Ar-H); 7.68-7.70(m,1H,Ar-H); 8.09-8.11(d,1H, Ar-H)；8.14-8.107 (m,2H, Ar-H); 9.81(s,1H,Ar-OH) | 484(M+2)  482(M+) |
| **5b** | 1.86-1.89(m,2H,COCCH_2_C;);2.18-2.22(m,2H,N(CCH_2_)C);2.56-2.62(m,2H,,NCCH_2_C);2.60-2.66(m,2H,COCH_2_);3.12-3.21(m,4H,N(CH_2_C)2);3.62-3.82(m,2H,COCCCH_2_);4.73(s,2H,N-CH_2_-Ar);5.76(s,H,OH);6.90-6.93(d,H,Ar-H);6.95-6.97(m,3H,Ar-H);7.35-7.39(m,2H,Ar-H);7.44-7.47 (d,2H,Ar-H); 7.72-7.74 (d,2H,Ar-H); 8.04-8.07 (m,2H, Ar-H); 9.81(s,1H,Ar-OH) | 484(M+2)  482(M+) |
| **5c** | 1.42(m,2H,COCCHC;);2.23-2.26(m,2H,N(CCH_2_)C); 2.59-2.64(m,2H,,NCCH_2_C);2.66-2.69(m,2H,COCH_2_);3.22-3.28(m,4H,N(CH_2_C)2);3.62-3.80(m,2H,COCCCH_2_);4.73(s,2H,N-CH_2_-Ar);6.01(s,1H,OH);;7.27-7.32(m,4H,Ar-H);7.55-7.59(m,2H,Ar-H);7.64-7.69 (m,3H,Ar-H); 7.79 (s,1H,Ar-H); 8.12-8.07 (m,2H, Ar-H); 9.79(s,1H,Ar-OH) | 484(M+2)  482(M+) |
| **6a** | 1.82(m,2H,CCH_2_C);2.26-2.50(m,4H,NCCH_2_CCH_2_C);3.33(t,2H,COCH_2_);3.54-3.77(m,6H,CH**_2_**N(CH_2_)_2_);4.88(s,2H,N-CH_2_-Ar);5.67(s,1H,OH);7.38-7.44(m,2H,Ar-H;2H,Ar-H);7.56-7.62(m,4H,Ar-H);7.69-7.71(m,1H,Ar-H);8.10-8.12(d,1H, Ar-H);8.13-8.15(m,2H, Ar-H); | 504(M+4)  502(M+2)  500(M +) |
| **6b** | 1.83(m,2H,CCH_2_C);2.24-2.44(m,4H,NCCH_2_CCH_2_C);3.37(t,2H,COCH_2_);3.45-3.65(m,6H,CH**_2_**N(CH_2_)_2_);4.79(s,2H,N-CH_2_-Ar);5.70(s,1H,OH);7.38-7.43(m,4H,Ar-H);7.56-7.58(m,2H,Ar-H);7.66-7.70(m,3H,Ar-H);7.87(s,1H,Ar-H); 8.10-8.14(m,2H,Ar-H); | 504(M+4)  502(M+2)  500(M +) |
| **6c** | 1.82(m,2H,CCH_2_C);2.23-2.43(m,4H,NCCH_2_CCH_2_C);3.32(t,2H,COCH_2_);3.43-3.66(m,6H,CH**_2_**N(CH_2_)_2_);4.78(s,2H,N-CH_2_-Ar);5.73(s,1H,OH);7.39-7.43(m,4H,Ar-H);7.51-7.53(m,2H,Ar-H;2H,Ar-H);7.72-7.75(m,2H,Ar-H);8.09-8.14(m,2H, Ar-H); | 504(M+4)  502(M+2)  500(M +) |
| **7b** | 1.62-1.65(m,2H,COCCHC);1.87-1.95(m,2H,N(CCH_2_)C);1.96-1.98(s,3H,COCH_3_);2.20-2.25(m,2H,,NCCH_2_C); 2.50-2.51(m,2H,COCH_2_);3.09-3.23(m,6H,N(CH_2_)3);3.27(s,2H,N-CH_2_-Ar);5.76(s,H,OH);7.33-7.36(m,2H,Ar-H);7.36-7.39(m,2H,Ar-H;m,3H,Ar-H; m,2H,Ar-H); 7.74-7.76 (S,H,Ar-H); 8.07-8.08 (m,2H, Ar-H); | 526(M+2)  524(M+) |
| **7c** | 1.63-1.65(m,2H,COCCHC);1.89-1.95(4H,N(CCH_2_)2C); 1.96-1.98(s,3H,COCH_3_);;2.57-2.59(m,2H,COCH_2_); 2.67-2.71(m,2H,,NCCH_2_C);3.48-3.51(m,6H,N(CH_2_)3); 3.56(s,2H,N-CH_2_-Ar);5.34(s,H,OH);7.07-7.11(m,2H,Ar-H); 7.25-7.27(d,2H,Ar-H); 7.42-7.47(m,4H,Ar-H);7.77-7.79 (d,2H,Ar-H); 8.11-8.14 (m,2H, Ar-H); | 526(M+2)  524(M+) |
| **8a** | 1.77-1.82(m,2H,COCCH_2_C);2.23-2.27,2,35-2.42(4H,N(CCH_2_)2C);2.49-2.51(m,2H,COCCCH);3.31-3.38(m,4H,N(CH_2_C)2);3.60-3.66(t,2H,COCH_2_);4.88(s,2H,N-CH_2_-Ar);5.71(s,H,OH);7.37-7.44(m,4H,Ar-H);7.46-7.48(d,2H,Ar-H);7.62-7.60(d,2H,Ar-H);7.78-7.80 (d,H,Ar-H);8.05-8.09 (s,H, Ar-H); 8.10-8.14 (m,2H, Ar-H); | 536(M+2)  534(M+) |
| **8b** | 1.78-1.81(m,2H,COCCH_2_C);2.22-2.26,2,35-2.43(4H,N(CCH_2_)2C);2.50-2.51(m,2H,COCCCH);3.23-3.34(m,4H,N(CH_2_C)2);3.50-3.57(t,2H,COCH_2_);4.85(s,2H,N-CH_2_-Ar);5.52(s,H,OH);7.40-7.45(m,4H,Ar-H);7.58-7.60(d,2H,Ar-H);7.79-7.83(s,2H,Ar-H);7.95-7.97 (s,H,Ar-H);8.09-8.13 (m,2H, Ar-H); 8.16 (s,H, Ar-H); | 536(M+2)  534(M+) |
| **8c** | 1.76-1.79(m,2H,COCCH^2^C);2.19-2.23,2,41-2.47(4H,N(CCH^2^)2C);2.50-2.51(m,2H,COCCCH);3.43-3.55(m,4H,N(CH^2^C)2);3.74-3.78(t,2H,COCH^2^);4.93(s,2H,N-CH^2^-Ar);5.48(s,H,OH);7.40-7.44(m,4H,Ar-H);7.56-7.58(d,2H,Ar-H);7.83-7.87(t,1H,Ar-H);7.91-7.95 (t,H,Ar-H);8.00-8.02 (m,2H, Ar-H); 8.10-8.14 (m,2H, Ar-H); | 536(M+2)  534(M+) |
| **9a** | 1.80(m,2H,CCH_2_C);2.26-2.47(m,4H,NCCH_2_CCH_2_C);2.51(s,3H,CH_3_-Ar);3.35(t,2H,COCH_2_);3.47-3.78(m,6H,CH**_2_**N(CH_2_)_2_);4.76(s,2H,N-CH_2_-Ar);5.61(s,1H,OH);7.34-7.38(m,1H,Ar-H); 7.39-7.45 (m,4H,Ar-H);7.42-7.45(m,2H, Ar-H); 7.58-7.61(m, 1H, Ar-H);7.61-7.65(m,2H, Ar-H); 8.11-8.16 (m,2H, Ar-H); | 482(M+2)  480(M +) |
| **9b** | 1.93(m,2H,CCH_2_C);2.24-2.68(m,4H,NCCH_2_CCH_2_C);2.38(s,3H,CH_3_-Ar);3.16(t,2H,COCH_2_);3.36-3.73(m,6H,CH**_2_**N(CH_2_)_2_);4.81(s,2H,N-CH_2_-Ar);5.89(s,1H,OH);7.35-7.39(m,3H,Ar-H);7.41-7.47(m,5H, ,Ar-H);7.74-7.77(d,2H, Ar-H); 8.04-8.05(m,2H ,Ar-H); | 482(M+2)  480(M +) |
| **9c** | 1.78-1.82(m,2H,CCH_2_C);2.20-2.24(m,2H,COCH2);2.34-2.41(m,4H,NCCH2CCH2C );3.28(S,3H, CH_3_); 3.43-3.50 (m,4H,N(CH_2_)_2_) ;3.58-3.64(m,2H. NCH_2_); 4.78(s,2H,N-CH_2_-Ar);5.73(s,1H,OH);7.36-7.38(m,4H,Ar-H);7.41-7.43(d,2H,Ar-H);7.55-7.57(d,2H,Ar-H);7.60-7.63(d,2H,Ar-H); 8.09-8.14(m,2H, Ar-H); | 482(M+2)  480(M +) |
| **10a** | 1.78-1.82(m,2H,COCCH_2_C);2.18-2.25,2,37-2.442(4H,N(CCH_2_)2C);2.49-2.51(m,2H,COCCCH);3.41-3.56(m,4H,N(CH_2_C)2);3.58-3.60(t,2H,COCH_2_);4.78(s,2H,N-CH_2_-Ar);5.54(s,H,OH);7.39-7.45(m,4H,Ar-H);7.46-7.48(d,2H,Ar-H);7.58-7.60(d,2H,Ar-H);7.62-7.69 (d,H,Ar-H);7.73-7.80 (s,H, Ar-H); 8.10-8.13 (m,2H, Ar-H); | 486 (M+2)  484 (M+) |
| **10b** | 1.78-1.82(m,2H,COCCH_2_C);2.18-2.25,2,35-2.442(4H,N(CCH_2_)2C);2.50-2.51(m,2H,COCCCH);3.48-3.53(m,4H,N(CH_2_C)2);3.58-3.64(t,2H,COCH_2)_;4.78(s,2H,N-CH_2_-Ar);5.66(s,H,OH);7.39-7.45(m,4H,Ar-H);7.46-7.48(d,2H,Ar-H);7.59-7.61(d,2H,Ar-H);7.76-7.68 (d,H,Ar-H);8.05-8.06 (s,H, Ar-H); 8.09-8.13 (m,2H, Ar-H); | 486 (M+2)  484 (M+) |
| **10c** | 1.78-1.82(m,2H,COCCH_2_C);2.18-2.25,2,37-2.442(4H,N(CCH_2_)2C);2.49-2.51(m,2H,COCCCH);3.415-3.59(m,4H,N(CH_2_C)2);3.59-3.62(t,2H,COCH_2_);4.78(s,2H,N-CH_2_-Ar);5.69(s,H,OH);7.39-7.45(m,4H,Ar-H);7.46-7.48(d,2H,Ar-H);7.57-7.60(d,2H,Ar-H);7.56-7.59 (d,2H,Ar-H); 8.10-8.13 (m,2H, Ar-H); | 486 (M+2)  484 (M+) |
| **11a** | 1.78-1.74(m,2H,COCCH_2_C);2.19-2.42(m,4H,N(CCH_2_)2C);3.27-3.29(m,2H,COCH_2_C);3.41-3.52(m,4H,N(CH_2_)2); 3.64-3.68(m,2H,CCH_2_N);5.11(s,2H,N-CH_2_-Ar);5.56(s,H,OH);7.39-7.44(m,4H,Ar-H);7.55-7.57(d,2H,Ar-H);7.88-7.92(m,H,Ar-H);7.95-8.02(m,2H,Ar-H);8.09-8.13(m,2H,Ar-H);8.23-8.25(d,1H,Ar-H) | 513(M+2)  511((M +) |
| **11b** | 1.79-1.83(m,2H,COCCHC);2.23-2.42(m,4H,N(CCH_2_)2C); 3.31-3.35(m,2H,COCH_2_)3.49-3.66(m,6H,N(CH_2_)3);4.93(s,2H,N-CH_2_-Ar);5.63(s,H,OH);7.39-7.44(m,4H,Ar-H);7.59-7.61(d,2H,Ar-H);7.85-7.89(m,H,Ar-H);8.09-8.16(m,3H,Ar-H);8.41-8.43(d,1H,Ar-H);8.63(S,1H, Ar-H). | 513(M+2)  511((M +) |
| **11c** | 1.94-2.03(m,2H,COCCHC);2.22-2.43(m,4H,N(CCH_2_)2C);3.30-3.35(t,2H,COCH_2_);3.49-3.56(m,6H,N(CH_2_)3);4.96(s,2H,N-CH_2_-Ar);5.77(s,H,OH); 7.32-7.40(m, 4H,Ar-H); 7.43-7.48(m,2H,Ar-H);8.01-8.06(m,2H,Ar-H);8.07-8.12(m,2H, Ar-H); 8.31-8.37(d,2H, Ar-H). | 513(M+2)  511((M +) |
| **12c** | 1.22-1.23(d,6H,C(CH_3_)2);1.89-1.92(m,2H,COCCHC);2.21-2.25(m,2H,N(CCH_2_)C);2.60-2.67(m,2H,,NCCH_2_C);2.92-2.99(m,2H,COCH_2_); 3.15-3.20(m,4H,N(CH_2_C)2); 3.20-3.22(m,1H,CH(C)2);3.63-3.69(m,2H,COCCCH_2_); 4.78(s,2H,N-CH_2_-Ar);5.78(s,H,OH);  7.34-7.45(d,4H,Ar-H); 7.46-7.51(m,4H,Ar-H); 7.73-7.75 (d,2H,Ar-H); 8.05-8.09 (m,2H, Ar-H) | 510(M+2)  508(M+) |
| **13b** | 1.78-1.82(m,2H,COCCHC;);2.21-2.25(m,2H,N(CCH_2_)C);2.35-2.41(m,2H,,NCCH_2_C);3.29-3.32(m,2H,COCH_2_);3.43-3.50(m,4H,N(CH_2_C)2);3.59-3.65(m,2H,COCCCH_2_);3.91(s,3H);4.83(s,2H,N-CH_2_-Ar);5.62(s,H,OH); 7.39-7.43(m,2H,Ar-H);7.58-7.60 (d,2H,Ar-H); 7.83-7.85 (d,2H,Ar-H); 8.09-8.13 (m,3H, Ar-H；s, H, Ar-H); | 526(M+2)  524(M+) |
| **13c** | 1.44-1.47(m,2H,COCCHC;);2.24-2.29(m,2H,N(CCH_2_)C);2.34-2.37(m,2H,,NCCH_2_C);2.96-2.99(m,2H,COCH_2_);3.28-3.29(m,4H,N(CH_2_C)2);3.48-3.53(m,2H,COCCCH_2_);3.89(s,3H);4.83(s,2H,N-CH_2_-Ar);5.52(s,H,OH); 7.32-7.38(m,4H,Ar-H);7.41-7.44 (d,2H,Ar-H); 7.56-7.58 (d,2H,Ar-H); 8.05-8.14 (m,4H, Ar-H); | 526(M+2)  524(M+) |
| **14a** | 1.74-1.81(m,2H,COCCH_2_C);2.20-2.24,2,35-2.41(4H,N(CCH_2_)2C);2.50-2.51(m,2H,COCCCH);3.14-3.16(m,4H,N(CH_2_C)2);3.58-3.62(t,2H,COCH_2_);4.88(s,2H,N-CH_2_-Ar);5.71(s,H,OH);7.37-7.43(m,4H,Ar-H);7.49-7.55(m,2H,Ar-H);7.81-7.90(m,H,Ar-H);7.98-8.02 (m,2H,Ar-H); 8.08-8.13 (m,2H, Ar-H); 8.16-8.20(m,1H, Ar-H). | 493(M+2)  491(M+) |
| **14b** | 1.73-1.81(m,2H,COCCH_2_C);2.22-2.28,2,35-2.41(4H,N(CCH_2_)2C);2.52-2.55(m,2H,COCCCH);3.14-3.16(m,4H,N(CH_2_C)2);3.58-3.62(t,2H,COCH_2_);4.88(s,2H,N-CH_2_-Ar);5.71(s,H,OH);7.37-7.41(m,4H,Ar-H);7.55-7.59(m,2H,Ar-H);7.81-7.88(m,H,Ar-H);8.01-8.08 (m,2H,Ar-H); 8.12-8.18 (m,1H, Ar-H); 8.18-8.22(d,1H, Ar-H).;8.54(S,1H, Ar-H) | 493(M+2)  491(M+) |
| **14c** | 1.74-1.82(m,2H,COCCH_2_C);2.19-2.23,2,34-2.41(4H,N(CCH_2_)2C);2.50-2.51(m,2H,COCCCH);3.15-3.17(m,4H,N(CH_2_C)2);3.55-3.64(t,2H,COCH_2_);4.87(s,2H,N-CH_2_-Ar);5.71(s,H,OH);7.37-7.43(m,4H,Ar-H);7.45-7.48(d,2H,Ar-H);7.60-7.62(d,2H,Ar-H);7.91-7.93 (d,2H,Ar-H); 8.08-8.13 (m,2H, Ar-H); | 493(M+2)  491(M+) |
| **15a** | 1.23-1.25(m,3H,Ar-C-CH_3_); 1.76-1.80(m,2H,COCCHC;); 2.18-2.22(m,2H,N(CCH_2_)C); 2.36-2.39(m,2H,,NCCH_2_C); 2.61-2.68(m,2H,COCH_2_);3.46-3.497(m,2H,Ar-CH_2_C);3.39-3.42(m,4H,N(CH_2_C)2);3.54-3.60(m,2H,COCCCH_2_);;4.65(s,2H,N-CH_2_-Ar);5.54(s,H,OH);7.37-7.44(m,4H,Ar-H);7.46-7.48(d,2H,Ar-H);7.62-7.60(d,2H,Ar-H);7.78-7.80 (d,H,Ar-H);8.05-8.09 (s,H, Ar-H);; 8.09-8.12 (m,2H, Ar-H); | 496(M+2)  494((M +) |
| **15c** | 1.18-1.22(m,3H,Ar-C-CH_3_);1.75-1.79(m,2H,COCCHC;);2.20(m,2H,N(CCH_2_)C);2.32-2.39(m,2H,,NCCH_2_C); 2.63-2.69(m,2H,COCH_2_);3.26-3.27(m,2H,Ar-CH_2_C);3.39-3.42(m,4H,N(CH_2_C)2);3.54-3.60(m,2H,COCCCH_2_);;4.65(s,2H,N-CH_2_-Ar);5.54(s,H,OH); 7.35-7.43(m,6H,Ar-H); 7.54-7.58 (m,4H,Ar-H); 8.08-8.11 (m,2H, Ar-H); | 496(M+2)  494((M +) |
| **16c** | 1.31(s,9H,C(CH_3_)3);2.33-2.51(m,2H,COCCHC;4H,N(CCH_2_)2C);3.12-3.15(t,2H,COCH_2_);3.48-3.51(m,4H,N(CH_2_C)2);4.153-4.212(t,2H,COCCCH_2_);4.66(s,2H,N-CH_2_-Ar);6.34(s,H,OH);7.07-7.11(m,2H,Ar-H);7.25-7.27(d,2H,Ar-H);7.42-7.47(m,4H,Ar-H);7.57-7.59 (d,2H,Ar-H); 7.93-7.96 (m,2H, Ar-H); | 524(M+2)  522(M+) |
