# Supplementary material for: Design, Synthesis, and Pharmacological Evaluation of Haloperidol Derivatives as Novel Potent Calcium Channel Blockers with Vasodilator Activity
Source: PLoS One. 2011 Nov 16;6(11):e27673. doi: 10.1371/journal.pone.0027673 (PMC3218019; doi:10.1371/journal.pone.0027673)
Supplement: Table S4 — A comparison of the sensitivity (IC50) to different compounds in endothelium-intact and -denuded thoracic aorta rings from rats. (DOC) [file pone.0027673.s004.doc]

Table S4. A comparison of the sensitivity (IC_50_) to different compounds in endothelium-intact and -denuded thoracic aorta rings from rats.

|  | Endothelium-intact | Endothelium- denuded |
| --- | --- | --- |
| Compound | IC_50_ (μM)* | IC_50_ (μM)* |
| **1** | 105.02±4.8 | 112±8.5 |
| **2** | 32.25±1.9 | 30.57±2.5 |
| **3** | 20.2±1.2 | 18.5±1.1 |
| **4** | 10.08±0.96 | 10.15±1.21 |
| **5a** | 12.95±1.21 | 13.11±1.33 |
| **5b** | 10.97±0.99 | 10.85±1.12 |
| **5c** | 10.06±1.03 | 9.99±0.98 |
| **6a** | 8.51±0.82 | 8.47±0. 77 |
| **6b** | 6.17±0.47 | 6.43±0.49 |
| **6c** | 5.01±0.41 | 5.21±0.69 |
| **7b** | 9.83±0.85 | 9.86±1.01 |
| **7c** | 6.15±0.52 | 6.55±0.82 |
| **8a** | 9.33±0.74 | 9.39±0.82 |
| **8b** | 4.37±0.32 | 4.26±0.31 |
| **8c** | 3.63±0.22 | 3.29±0.31 |
| **9a** | 8.51±0.83 | 8.44±0.72 |
| **9b** | 6.31±0.42 | 6.45±0.57 |
| **9c** | 5.37±0.61 | 5.42±0.79 |
| **10a** | 9.55±0.78 | 9.57±0.93 |
| **10b** | 8.32±0.68 | 8.17±0.85 |
| **10c** | 8.51±0.72 | 8.59±0.68 |
| **11a** | 10.72±0.88 | 10.69±1.27 |
| **11b** | 7.24±0.63 | 7.28±0.59 |
| **11c** | 5.37±0.42 | 5.41±0.65 |
| **12c** | 2.26±0.53 | 2.48±0.45 |
| **13b** | 5.42±0.62 | 5.98±0.59 |
| **13c** | 3.56±0.41 | 3.66±0.52 |
| **14a** | 19.05±1.36 | 18.94±1.44 |
| **14b** | 11.75±1.02 | 11.59±1.12 |
| **14c** | 8.13±0.68 | 8.04±0.61 |
| **15a** | 6.92±0.46 | 6.69±0.58 |
| **15c** | 2.63±0.28 | 2.51±0.32 |
| **16c** | **0.95±0.072** |  |
