# Supplementary material for: Design, Synthesis, and Pharmacological Evaluation of Haloperidol Derivatives as Novel Potent Calcium Channel Blockers with Vasodilator Activity
Source: PLoS One. 2011 Nov 16;6(11):e27673. doi: 10.1371/journal.pone.0027673 (PMC3218019; doi:10.1371/journal.pone.0027673)
Supplement: Table S5 — 1HNMR data of Compounds I–V. (DOC) [file pone.0027673.s005.doc]

Table S5. ^1^HNMR data of Compounds **Ⅰ-Ⅴ**

| Compounds | ^1^HNMR/300 MHz, CDCl_3_-d_1_/DMSO-d_6_ |
| --- | --- |
| Ⅰ | 7.33-7.39（m,1H, Ar H）,7.17-7.24 (m, 3H, Ar H) 4.52 (s, 2H, CH_2_)，2.31 (s, 3H, CH_3_). |
| Ⅱ | 7.43 (d, 2H, Ar H), 7.04 (d, 2H, Ar H), 4.52 (s, 2H, CH_2_), 2.26 (s, 3H, CH_3_). |
| Ⅲ | 4.68 (s, 2H, CH_2_)，6.76-6.78（d,1H,ArH）, 6.79-6.85(m,2H,ArH), 7.13-7.17 (m,1H,ArH)，7.27-7.29（(d,2H,ArH),9.76(s,1H,ArOH) |
| Ⅳ | 4.65 (s, 2H, CH_2_)，6.69-6.73（d,1H,ArH）, 6.82-6.83(m,2H,ArH), 7.09-7.17 (m,1H,ArH).9.42(s,1H,ArOH) |
| Ⅴ | 4.70 (s, 2H, CH_2_)，6.49-6.74(m,2H,ArH),6.83-7.04((m,2H,ArH),9.75(s,1H,ArOH) |
