# Supplementary figures and images for: Design, Synthesis, and Pharmacological Evaluation of Haloperidol Derivatives as Novel Potent Calcium Channel Blockers with Vasodilator Activity
Source: PLoS One. 2011 Nov 16;6(11):e27673. doi: 10.1371/journal.pone.0027673 (PMC3218019; doi:10.1371/journal.pone.0027673)

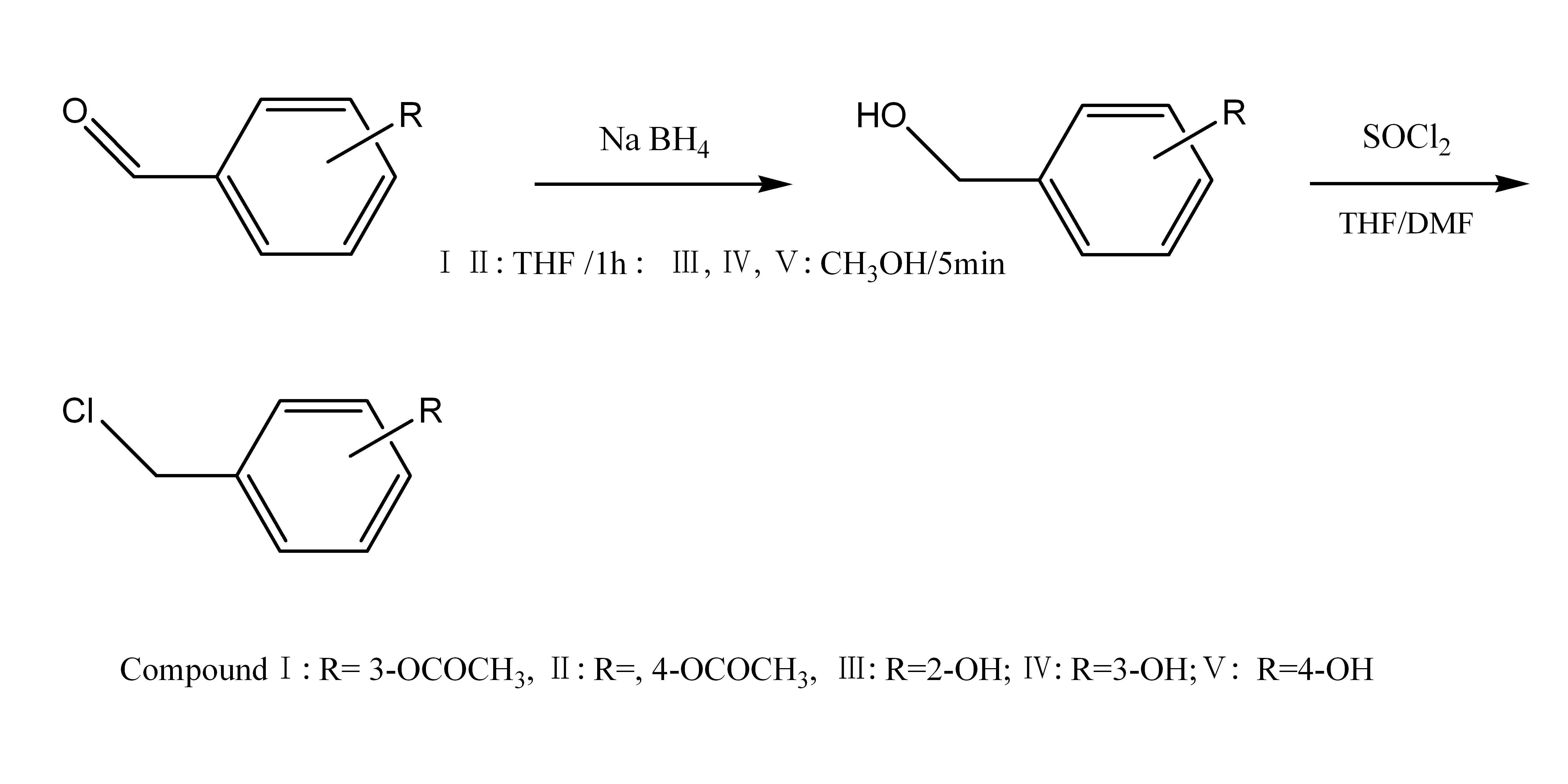

Supplement: Scheme S1 — Reagents and conditions. (a) NaBH4, MeOH, room temperature 5 min, or NaBH4, THF, room temperature 1 hour. (b) SOCl2, THF/DMF, ice-bath 20 min. (TIF) [file pone.0027673.s006.tif]

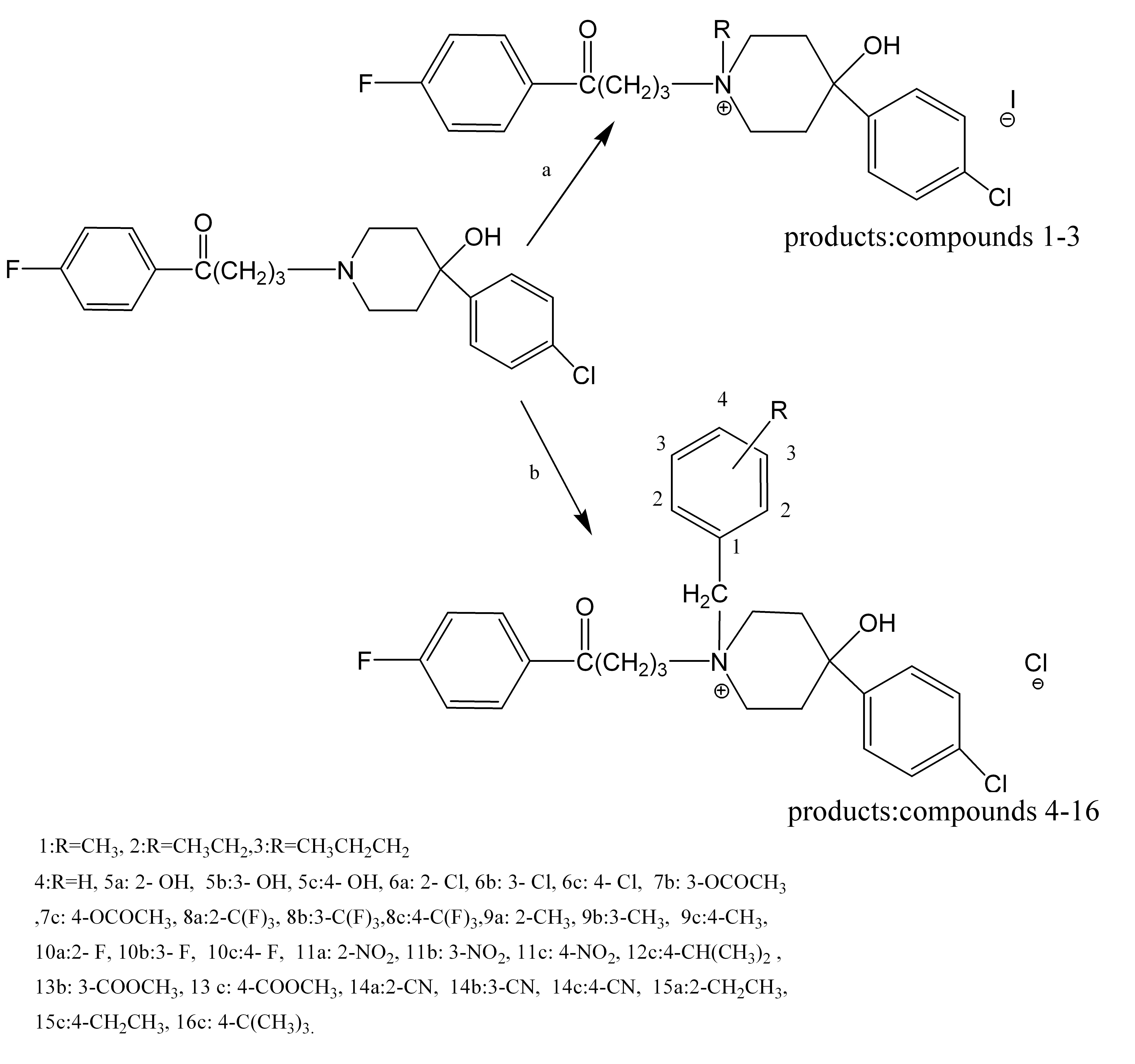

Supplement: Scheme S2 — Reagents and conditions. (a) CH3I or CH3CH2I or CH3 (CH2)2I, reflux, 24 h. (b) The benzyl halidel, CHCl3, reflux, 18–24 h. (TIF) [file pone.0027673.s007.tif]
